# The lesson learned from COVID-19 and the climate crisis is not to let experts decide on policies: a response to Robert C. Schmidt

**DOI:** 10.1007/s13412-021-00737-7

**Published:** 2021-11-27

**Authors:** Annette Elisabeth Toeller, Sonja Blum, Michael Boecher, Kathrin Loer

**Affiliations:** 1 Chair for Policy Research & Environmental Politics, FernUniversitaet in Hagen, Universitaetsstr. 33, 58084 Hagen, Germany; 2 Chair for Political Science and Sustainable Development, Otto-Von-Guericke-Universitaet Magdeburg, Universitaetsplatz 2, 39106 Magdeburg, Germany; 3 Chair for Political Science, Hochschule Osnabrueck, Caprivistraße 30a, 49076 Osnabrueck, Germany

**Keywords:** Climate change, Pandemic, Democracy, Science, Politics

## Abstract

This is a response to the commentary by Robert C. Schmidt in this journal, in which the author suggests that for specific problems such as climate change or the current pandemic, decisions on policies should be made by scientific experts rather than by politicians. We argue that such ideas, which were brought up in the late 1960s and reconsidered more recently, do not take sufficient account of the nature of science politics, and their interaction. Furthermore, problem structures and resulting challenges for science and politics are not similar, but essentially different between climate change and the pandemic. Therefore, different solutions to the problems are required. There is a need to improve politics’ reliable recourse to scientific evidence in many cases. Yet, giving scientific experts such a strong position in decision-making ignores that most decisions, even if based on the state of scientific evidence (if there *is* such an uncontroversial state of evidence), ultimately require genuinely political choices about trade-offs of interests and normative issues that neither can nor should be made by scientists. Therefore, putting Schmidt’s proposal into practice would not solve the existing problems but instead create new problems.

## Introduction

In his contribution, Robert C. Schmidt argues that there are major similarities between the COVID-19 crisis and the climate crisis. In addition to uncertainty and free-rider problems, the core of his diagnosis is that there is a “disincentive of politicians to adequately address the respective issue,” which leads to their inability to adopt “early, farsighted, and possibly harsh policy measures.” As a consequence, he suggests “changes in the political system itself.” For “complex problems with certain characteristics,” he recommends “novel political decision procedures that sidestep the normal, day-to-day political proceedings,” which “actively involve experts and lower the involvement of political parties as far as possible to minimize the decision-makers’ disincentives” (Schmidt 2021).

In this response, we argue that insights from political science research are relevant to the issues raised by Schmidt. They demonstrate both that some of his findings are questionable, and that the proposals he formulates have problematic implications in various respects. As political scientists in the fields of environmental politics, public policy, and public health, as well as members of scientific advisory bodies to the German federal government, we also wish to contribute to broader debates about a meaningful division of labor between science and politics in the design of public policies.

In the following, we will first reflect on the problems of democratic systems to handle major challenges, such as the climate crisis, thereby confirming core points of Schmidt’s problem diagnosis. We then turn to the relationship of science and politics, and then compare the problem structures of two major crises of our time—COVID-19 and the climate crisis. Based on this analysis, we conclude why side-stepping politics will not help, and propose some alternative ideas.

## The performance of democracy in addressing major challenges

Robert C. Schmidt’s commentary addresses, on a general level, the suitability of democratic systems to deal with major challenges such as climate change, biodiversity loss, plastic pollution, or epidemics. In fact, the question of whether democracies are capable of adopting and implementing effective solutions to major challenges facing humanity and the planet has been going on for decades. Starting with discussions in the late 1960s and 1970s (e.g., Hardin 1968; Ophuls 1977), the idea that the democratic constitutional state is incapable of taking sufficient, timely, and appropriate policy action, particularly with regard to environmental challenges in general and climate change in particular, has become popular more recently (e.g., Shearman and Smith 2007; cf. Held and Hervey 2009; Dobson 2010; Jamieson 2020). However, these debates demonstrate that it would be simplistic to focus on the motivational structure of party politicians only. Rather, the causes of the identified shortcomings are complex and multifaceted.

Indeed, election cycles of 4 to 5 years do not help politicians in pursuing policies for the sake of long-term goals (Held and Hervey 2009; Povitkina 2020). In addition, as also addressed by Schmidt, there is a problem in the representation of societal interests: business interests are usually better able to assert their positions vis-à-vis politicians than, for instance, environmental or consumer interests (Olson 1965). Moreover, the interests of future generations, who will feel the consequences of climate change to a much stronger extent than we do today, are not represented in the political process either as voters or as stakeholders (e.g., SRU 2019). In addition, voters still give environmental issues little consideration, and there is a gap between environmental awareness and individual willingness to accept costs for better environmental quality (e.g., Tobler et al. 2012). As a result, political representatives, who ultimately depend on being (re-)elected, find it difficult to raise environmental issues in the political process, especially when, as with climate change policy, the benefits of costly measures can only be expected to materialize in the far future (Povitkina 2020).

Further restrictions on the free choice of policies result from a liberal concept of individual freedom, which is expressed in constitutional principles such as the protection of property and individual fundamental rights and freedoms, as well as the principle of proportionality, which severely limit what governments can do in most democracies, albeit to different degrees. A problem is the weakness of international institutions, since many ecological problems are global in nature and can only be addressed through globally coordinated action (Held and Hervey 2009: 9; Jamieson 2020).

In the face of all these problems, several authors argue that democracy must be (partly) sacrificed to enable sustainable solutions (e.g., Hardin 1968; Ophuls 1977). More recently, Lovelock (2010) argued that due to the severity of climate change, it may at least “be necessary to put democracy on hold for a while” (Lovelock 2010; for a discussion, see Dobson 2010), and Shearman and Smith (2007) hold that a technocratic, authoritarian form of government is necessary.

However, since the environmental performance of authoritarian regimes does not seem to be better than that of democratic regimes (Held and Hervey 2009: 6–7; Bättig and Bernauer 2009; Povitkina 2020), promising solutions aim not at abolishing democracy as such, but at “optimizing” decision-making within democracies. In particular, this involves institutional brakes on majority rule (e.g., by anchoring climate protection in the constitution, and establishing councils, e.g., for intergenerational justice, which can exert influence via proposal rights and (suspensive) veto rights, e.g., SRU 2019: 180), and giving courts as well as science a stronger role in politics. The latter point is proposed by Robert C. Schmidt in his commentary, and we will now turn to it in more detail.

## Science and politics: general concepts

Schmidt suggests that when it comes to decision-making on major challenges, scientific experts should shape political decisions more directly and quickly—without the delaying and distracting influences of politicians and political processes in democracies. We argue that this claim—albeit common—ignores both what we know about the logic of science and politics, as well as considerations of democratic legitimacy.

First, it is far from trivial to state that politics is not science, and science is not politics. Caplan (1979) captured this argument in the image of the “two communities,” and argued that scientists and policy makers act in completely different worlds, based on different interests and values (Böcher 2020). As also stressed by Schmidt’s commentary, politics follows the logic of acquiring power and pursuing interests under short-term conditions, and is thus entirely different from science, which at its core is characterized by the continuous, in principle unlimited search for truth. And as a consequence, indeed, scientific evidence^1^ (no matter how valid and reliable) remains (too) often ignored or unused politically, if it is not in line with the interests of powerful political actors. Yet this fundamental problem cannot be solved by making scientists act as politicians (Böcher and Krott 2016). On the contrary, given these fundamental differences between the two spheres, it is crucial to respect the boundaries between scientific and political actors. For scientific experts, this implies that they must make transparent which of their arguments are science-based and which are not. Otherwise, they risk a confusion of scientific evidence and political opinions, which can damage the authority of science-based arguments, which might then appear just as debatable opinions.

Emphasizing that the basic logic of science—as opposed to politics—is the search for truth does also not imply that the world of science itself is a world without interests and power. First, science is also the result of and in turn reproduces social power relations, in particular with regard to socioeconomic, racial, and gender relations. Second, already the choice of experts is a political exercise, even though the extent of politicization may vary between countries and even within countries from one expert body to the other. Third, science and scientific experts, too, are socially and economically embedded, dependent on gaining resources, and interested in dominating discourses. Especially if scientific organizations are strongly involved in “evidence-based policymaking” as is particularly the case in the field of public health, they can be prone to commercialization and politicization, as the example of ILSI (“International Life Science Institute”) demonstrates (Strassheim and Loer 2019). Thus, to portray science as a neutral and apolitical exercise (and therefore as the better alternative to politics) is far from reality.

Moreover, the idea that stronger expert involvement inevitably improves decision-making results not only ignores these different logics of science and politics. It is also reminiscent of outdated linear (or technocratic) models of scientific policy advice, according to which scientific expertise can lead directly to political decisions. This would imply that the more scientists produce evidence related to policy problems, the more intensively it translates into science-based policy decisions (Hulme 2009). Albeit still being discussed, such linear models display a number of weaknesses. The first weakness is the assumption that there is always one undisputed scientific diagnosis of a problem. In real life, especially in environmental politics, controversies over scientific problem definitions are the rule rather than the exception (Beck 1986). Moreover, there are usually different disciplines that have a contribution to make, both on problem definitions and possible solutions. Hence, there usually is not “one” scientific evidence but rather a variety of (often competing) evidences that has to be dealt with and needs to be integrated if it is to inform political decision-making (Schoemaker et al. 2020). Furthermore, even a clear scientific problem definition does not automatically produce a “first best” policy solution to solve the problem. Rather, the choice for one or the other policy option always implies decisions over values which tend to be not within the realm of scientific evidence (Hertin 2020).

Acknowledging that there can be no linear transfer of scientific knowledge into policies (Greenhalgh and Wieringa 2011; Böcher and Krott 2016; Sokolovska et al. 2019), several authors have argued that the interaction of science and politics must be conceptualized in a more complex way. For instance, the Research-Integration-Utilization (RIU) model emphasizes that—between the spheres of knowledge production and political decision-making—a sphere of integration is needed (Böcher and Krott 2016). Integration means that selection activities must occur in both directions. On the one hand, the demand of political actors for scientifically based solutions is identified and used to select relevant scientific findings. On the other hand, research results are interpreted, mediated, and selected according to their relevance for political actors. Thus, integration connects scientific findings with expectations and needs of political actors, yet without changing the scientific rationality of the research (Böcher 2016, 2020).

Finally, having scientific experts shape political decisions more directly while significantly reducing the role of politicians raises serious problems of democratic legitimacy. While politicians are democratically elected and can be held accountable by voters for their decisions in the coming election, this mechanism to secure political responsibility is missing in the case of expert decisions. Scientists can be recalled from their respective expert group, but they cannot be held accountable by citizens for decisions made on the basis of their expertise. This fundamental problem cannot be mitigated by somehow linking expert decisions to varieties of alternative decision-making procedures like citizens’ assemblies or referendums, as Schmidt suggests (Schmidt 2021).

## Science and politics: comparing the COVID-19 and climate crisis

In recent public discourses, some observers emphasize that in the pandemic, we witness an amazing recognition of scientific evidence both on part of citizens and politics (Hertin 2020). Others see unusually strong state interventions resulting in considerable restrictions of personal liberties (e.g., regarding air travel) and ask why similarly restrictive policies are not implemented to fight climate change (e.g., Povitkina 2020).

Schmidt, on the contrary, emphasizes similarities between the two “crises”: according to him, the most important similarity lies in the time-lag between “efforts” and the “benefits resulting from these efforts.” As we will argue, however, differences between the COVID-19 crisis and the climate crisis prevail, as well as in their implications for science-policy interaction.

To begin with, there is a certain time-lag between cause and possible effects in *all* imaginable policy measures (with very few exceptions). Yet between climate policy and infection-control measures under COVID-19, the scale of the time-lag could in fact not differ more: decades to centuries in one case, days to weeks in the other. Moreover, the causal link between policies and their effect is also much more indirect and uncertain in the case of climate change, and more direct in fighting the pandemic. Accordingly, the “urgency” for policy intervention varies. Of course, in climate change, political action cannot be postponed till “doomsday.” Yet in the pandemic, developments sometimes require weekly readjustments of policies. This dissimilarity is crucial, since the “time dimension” of a problem has been identified as a key factor by research, distinguishing long-term/anticipatory from short-term/reactive policy contents, and arguing how those come along with different advisory actors and formats (Craft and Howlett 2010). In addition, the pandemic concerns the classical tasks of the (nation) state to protect the lives of the citizens on its territory, whereas this is not the case to the same extent with climate change, especially for most countries in the North.

We agree with Schmidt that the dependence on a scientific interpretation of the problem as well as of possible solutions is what the two crises do have in common. Yet when looking at the “first wave” of the pandemic arriving in most countries in early 2020, the political “adherence” to scientific knowledge was in fact exceptionally strong. While for the climate crisis, scientific knowledge is in many respects available (although incomplete), it was—at least in spring 2020—in many respects still rather patchy and provisional for the case of COVID-19. Nonetheless, and *before* the pandemic effects even fully materialized (e.g., with high national infection numbers or death tolls), most countries enacted strict and unprecedented containment policies, such as curfews, business closures, or childcare and school closures (see for an overview of measures: Hale et al. 2021). Governments did take preventive action, following scientific advice, and general epidemiological models of “flattening the curve” and “the hammer and the dance” (see e.g. Pueyo 2020). Politics also *needed* to decide on a multitude of matters on which the scientific knowledge was still lacking, unclear, or disputed (see e.g. Mallapaty 2020).

Comparing the COVID-19 and the climate crisis may also serve to illustrate some points, which we argue to be of generalizable importance for the science-politics relationship.

The pandemic childcare and school closures demonstrate that different disciplines are needed to arrive at holistic policy solutions—here, among others, including pedagogy or social scientists next to public-health experts. Similarly, effectively addressing climate change requires not only the expertise of natural scientists, but also that of e.g. economists, historians, or political scientists. Since their disciplinary view on policy measures would differ, with view to Schmidt’s proposal of a “scientific” decision-making, again the fundamental question arises of who should *select* those experts and who would provide for an integration of different bodies of evidence.

Gaining more (scientific) information can reduce uncertainty, but it cannot reduce the ambiguity that is inherent to political decisions (Kingdon 1984). Even on climate change, where there is now a 97% scientific consensus on its causes and consequences, there is persistent controversy among scientists about preferable solutions. Which instruments are best suited to reduce greenhouse gas emissions? Market-based instruments like taxes, certificate trading, or stricter command-and-control regulation? Which instruments are not only technically viable, economically efficient, and socially just, but also politically realizable? In the case of the pandemic, when it became clear that children, too, have a role for disease transmission, this did not mean that the issue became less disputed: should schools *still* reopen (or not), because of their societal importance? And if yes, should other areas (and which) be regulated more strictly to reach the same containment potential overall? Scientific evidence does often not suffice as an unambivalent basis for making such political decisions. Scientific knowledge on the pandemic is as dynamic as the pandemic itself, and thus may only cover short periods of time, whereas citizens are tired of short-term decisions on lock-downs and other restrictions and long for being able to plan their lives again. While integrating multi-disciplinary and state-of-the art expertise is crucial to arrive at decisions, evidence can never fully back a political decision. This is a major reason why governments must be held politically accountable for political decisions, while science cannot (and should not) take this responsibility.

What is more, science cannot dissolve the consequences of policy action for different groups and the resulting conflicts that politics has to handle. In this regard, we see major differences in the underlying problem structures of climate change and COVID-19. Climate protection is primarily about protecting the “others” (future generations, island states), whereas “we” (current generations, affluent states) have to pay the bill. Contrariwise, for the pandemic, although there are different degrees in the need of protection (e.g., the old and the sick being at-risk groups) and different degrees in who bears the cost of restrictive measures, it is ultimately about self-protection (as there are severe courses of the disease in the young and healthy as well). This brings about different lines of conflict and challenges for political action. In both cases, it is not just a matter of implementing the “right” policies, but of balancing different interests in society. In both cases, the social (and racial) dimension of affectedness—by climate change, the pandemic, but also by the policies enacted to combat both—has not been adequately addressed so far. Yet, there are signs of change. For instance the prioritization of environmental policy instruments in the economic debate was for a long time primarily based on effectivity and efficiency considerations based on economic modelling. However, while the discussion on environmental justice in Germany is clearly underdeveloped—especially in comparison with the USA—the current discussion on climate change indicates a different focus. In the recent discussion on the necessity of more effective climate policy measures, relevant authors in science (and, incidentally, increasingly in politics) claim that “combating the climate crisis can go hand in hand with measures against poverty and social inequality” (Gründinger et al. 2021, translated from the authors) and the debate has just arrived in German media (e.g., Frondel et al 2021). Yet, with regard to climate change, a major balance must be struck between current and future generations. A balance between strong and weak societal interests is central with regard to COVID-19, too. For example, business continues to have effective mouthpieces, while so-called weak interests find it difficult to make themselves heard. This applies both to groups that are vulnerable with regard to the virus (the elderly, the chronically ill, caregivers, and also the less privileged) and to those who suffer particularly from restrictive measures (children, women and children in precarious circumstances, the mentally ill, etc.).

## Conclusion

Taking important decisions about the future, such as on climate mitigation or the protection of public health in a pandemic, out of (party) political decision-making and handing them—to a certain degree—over to experts, as Robert C. Schmidt proposes, is in our view neither a workable nor a legitimate solution to existing problems.

First, the idea is based on a linear, outdated, and unrealistic understanding of how science and politics work. In fact, the two follow different logics. Therefore, the boundaries between science and policymaking must be respected while structuring their interaction in a meaningful way. Commissioning science to make policy decisions would necessarily require action beyond established evidence sooner or later. While this is precisely the mandate of elected politicians, scientists not only lack the necessary legitimacy—it would also compromise scientists, since they receive their authority through sound evidence obtained according to the strict criteria of producing scientific knowledge. It could ultimately call scientific authority into question and, as a result, pour fuel on the fire of populists who fundamentally question the legitimacy of scientific authority.

Second, whereas some restrictions on effective policies, both against climate change and the pandemic, indeed result from the logic of democratic decision-making, others stem from the principles of constitutional democracy. This would be a barrier to expert decision as much as it is to political decision-making, and rightly so because it protects the fundamental rights of citizens. No end justifies all means.

Third, who exactly is to decide which decisions are to be taken out of the hands of politics? Schmidt suggests problems like climate change, insect extinction, plastic pollution, or pandemics. We would agree that these are major problems, but others might define famine or social and race inequality as the major problems that need to be effectively addressed by experts instead of democratic politics.

Fourth, the challenges in combating climate change and the COVID-19 pandemic are essentially different in nature. On climate change, there is now, in many respects, established scientific evidence, as there are institutions and processes to make that evidence available to policymakers. The challenge here is to take targeted measures now. More purposeful, consistent action can be achieved by strengthening the societal representation of future generations (the rise of the Fridays for Future or similar movements in many countries is a very important development in this regard). This includes adapting institutional arrangements in democratic decision-making processes, while not neglecting a fair balance within present generations. With regard to the pandemic, the aim must be, first, to reliably integrate the current, very dynamically developing state of scientific evidence from very different disciplines into the policy process. Instead of drawing on scientific experts in a random and selective way (as seems sometimes to be the case e.g. in Germany) and thus risking to instrumentalize science for those positions that seem politically suitable, politics should institutionalize advice by establishing a council of experts on pandemics, as they exist in some countries as well as in other policy sectors. Such councils indicate that these are reliable experts from a relevant range of disciplines whose positions should be taken into account. It is important to raise awareness of the fact that virological, epidemiological, etc. findings do not translate “naturally” and unambivalently into policies. Instead, such findings must be considered in the context of other, e.g., sociological, educational, or psychiatric findings. The experts themselves should have to integrate their different positions and translate them into digestible products that politicians can rely on. Establishing scientific councils does not imply that these experts should *decide* on policies. Decisions on policy measures always require the balancing of different social interests in specific national or regional contexts. There are better and worse variants of pandemic management, but there is not “one ideal policy.”

Fifth, in a democratic society, one must persevere that no one (not even experts on climate change or public health) has a monopoly on determining the public definition of problems. Even legitimate advocates of severe and eminently important problems must face political competition over the important problems and the right solutions. Advocates of rigorously addressing climate change for the benefit of future generations must also face demands for equity and fairness in today’s world, and supporters of rigid measures to address the global pandemic have to answer questions about the impact of those rigid measures on members of vulnerable groups.

There are no simple solutions to complicated problems.

## Footnotes

1 When using the term “evidence” here, we include all sorts of results of scientific research that have been produced according to the state-of-the-art methods of the respective discipline. We do not restrict the concept of evidence to results of tests in a controlled setting as is the standard in some disciplines and thus might be associated with evidence by some observers. We are grateful to the reviewer who pointed to these different ways to understand scientific evidence.
